# Supplementary material for: Comparisons of the antibody repertoires of a humanized rodent and humans by high throughput sequencing
Source: Sci Rep. 2020 Jan 24;10:1120. doi: 10.1038/s41598-020-57764-7 (PMC6981180; doi:10.1038/s41598-020-57764-7)
Supplement: Supplementary file 1 — Supplementary Information. [file 41598_2020_57764_MOESM1_ESM.pdf]

SUPPLEMENTARY INFORMATION

TITLE: Comparisons of the antibody repertoires of a humanized rodent and humans by high throughput sequencing

AUTHORS: Collin Joyce[1,2,3,5], Dennis R. Burton[1,2,3,4,6,*] and Bryan Briney[1,2,3,4,5,*]

AFFILIATIONS:
[1]Department of Immunology and Microbiology, The Scripps Research Institute, La Jolla, CA, USA
[2]Consortium for HIV/AIDS Vaccine Development (CHAVD), The Scripps Research Institute, La Jolla, CA, USA
[3]IAVI Neutralizing Antibody Center, The Scripps Research Institute, La Jolla, CA, USA
[4]Human Vaccines Project, New York, NY, USA
[5]Center for Viral Systems Biology, The Scripps Research Institute, La Jolla, CA, USA
[6]Ragon Institute of MGH, MIT and Harvard, Cambridge, MA, USA

[*]To whom correspondence should be addressed.
Dennis R. Burton: burton@scripps.edu
Bryan Briney: briney@scripps.edu

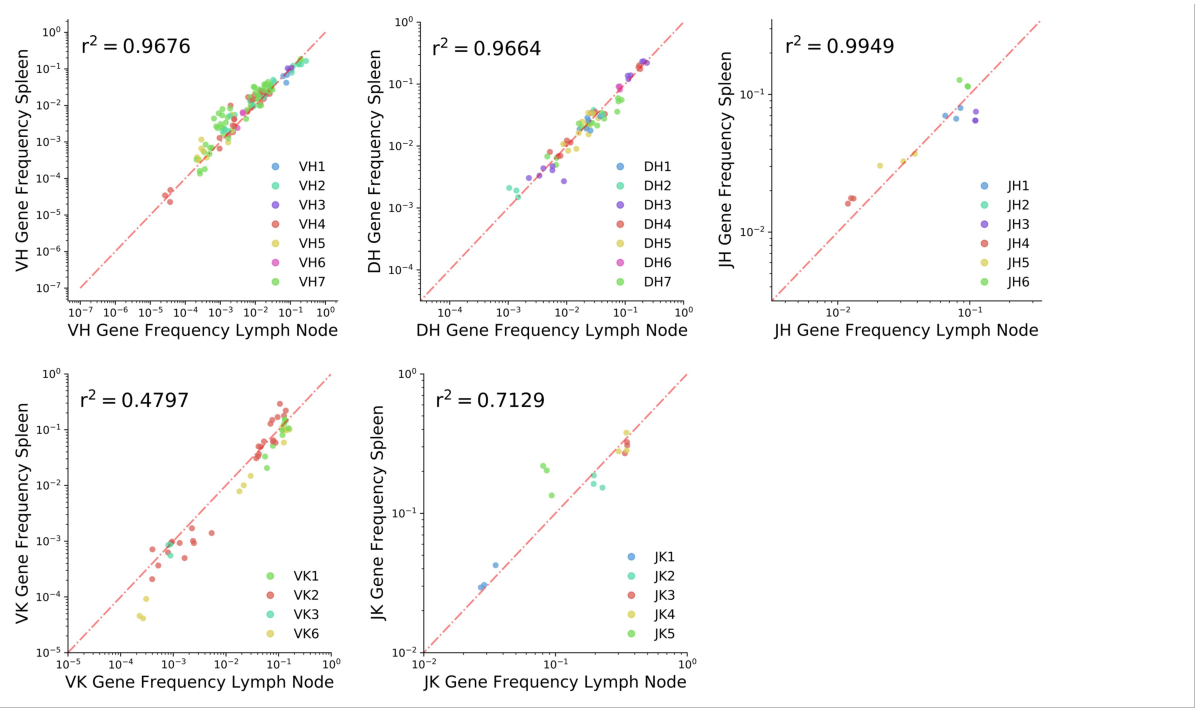

Supplementary Figure 1. Intra-animal gene segment usage frequency scatter plots.

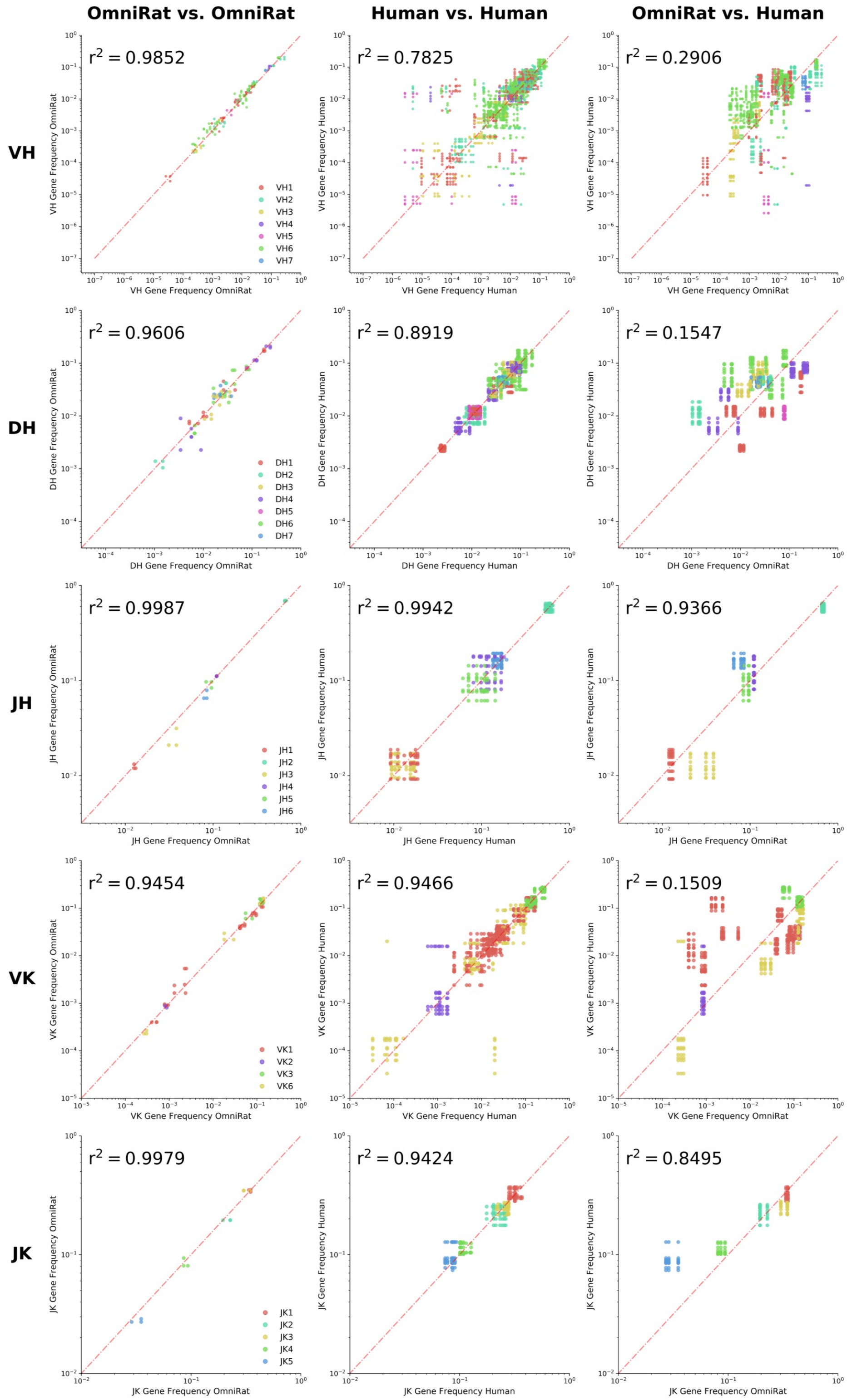

Supplementary Figure 2. Intra-species and inter-species gene segment usage frequency scatter plots.

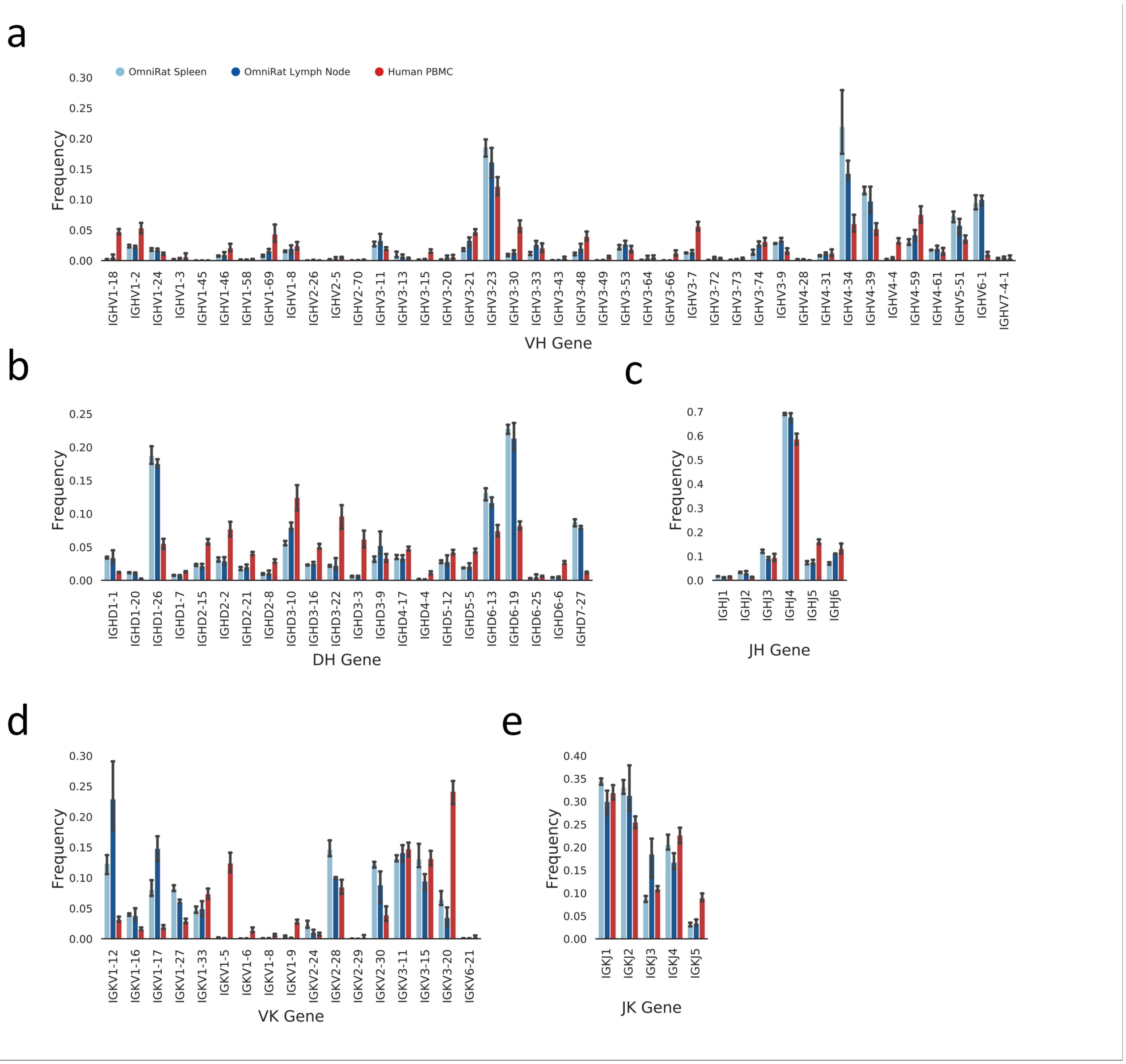

Supplementary Figure 3. Gene segment usage segment frequencies. for a) VH, b) DH, c) JH, d) VK, e) JK. Species are colored for all figures as in (a)

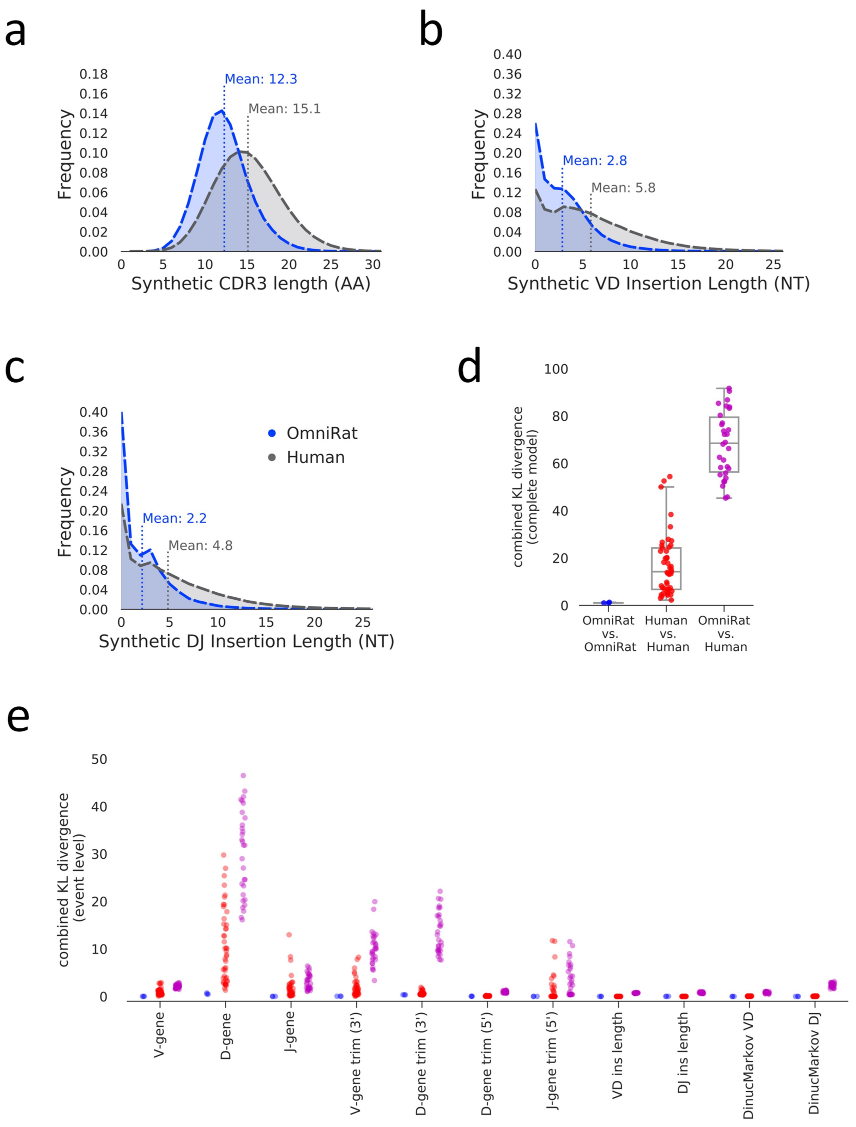

Supplementary Figure 4. Naïve Repertoire modeling with IGoR. a) Synthetic CDRH3 length distribution for each species. Species are colored as in (c). CDRH3 lengths were determined using the ImMunoGeneTics (IMGT) numbering scheme. b) Synthetic VD insertion length distributions for each species. Species are colored as in (c). c) Synthetic DJ insertion length distributions for each species. d) Combined Kullback–Leibler divergence (KL divergence) between pairs of OmniRat models (blue), between pairs of human models (red), or between pairs of OmniRat and human models (purple).

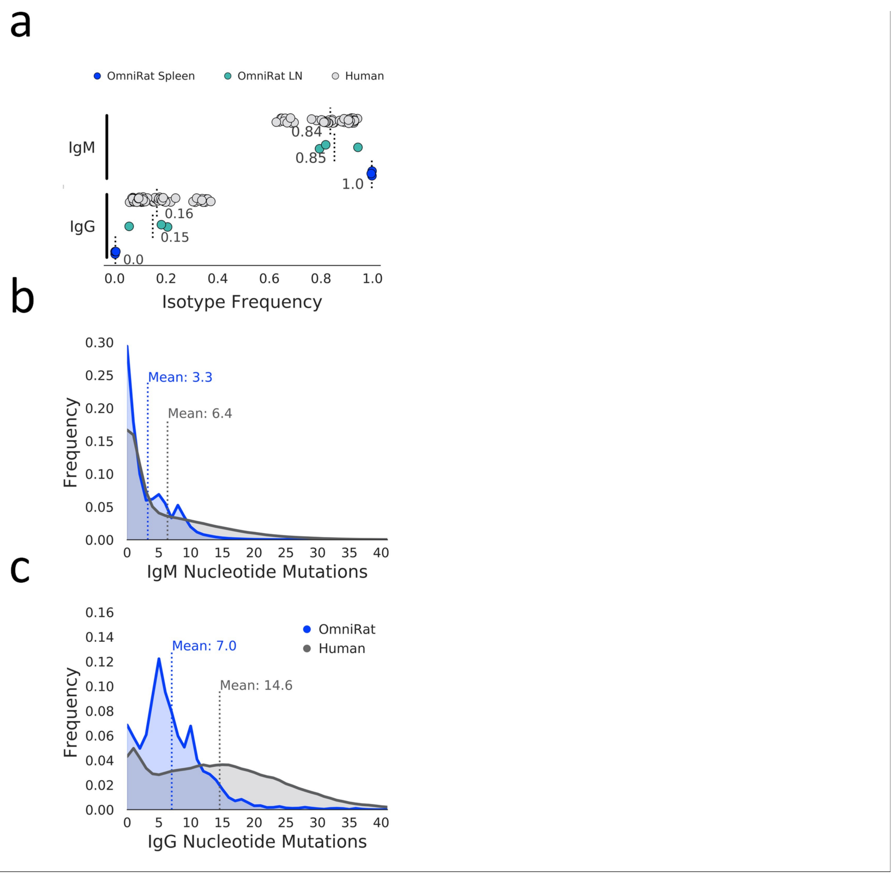

Supplementary Figure 5. Class-switch recombination and somatic hypermutation. a) Sequence frequency by antibody isotype. b) Variable heavy gene nucleotide mutation number distributions for IgM sequences. Species colored as in (c). c) Variable heavy gene nucleotide mutation number distributions for IgG sequences.

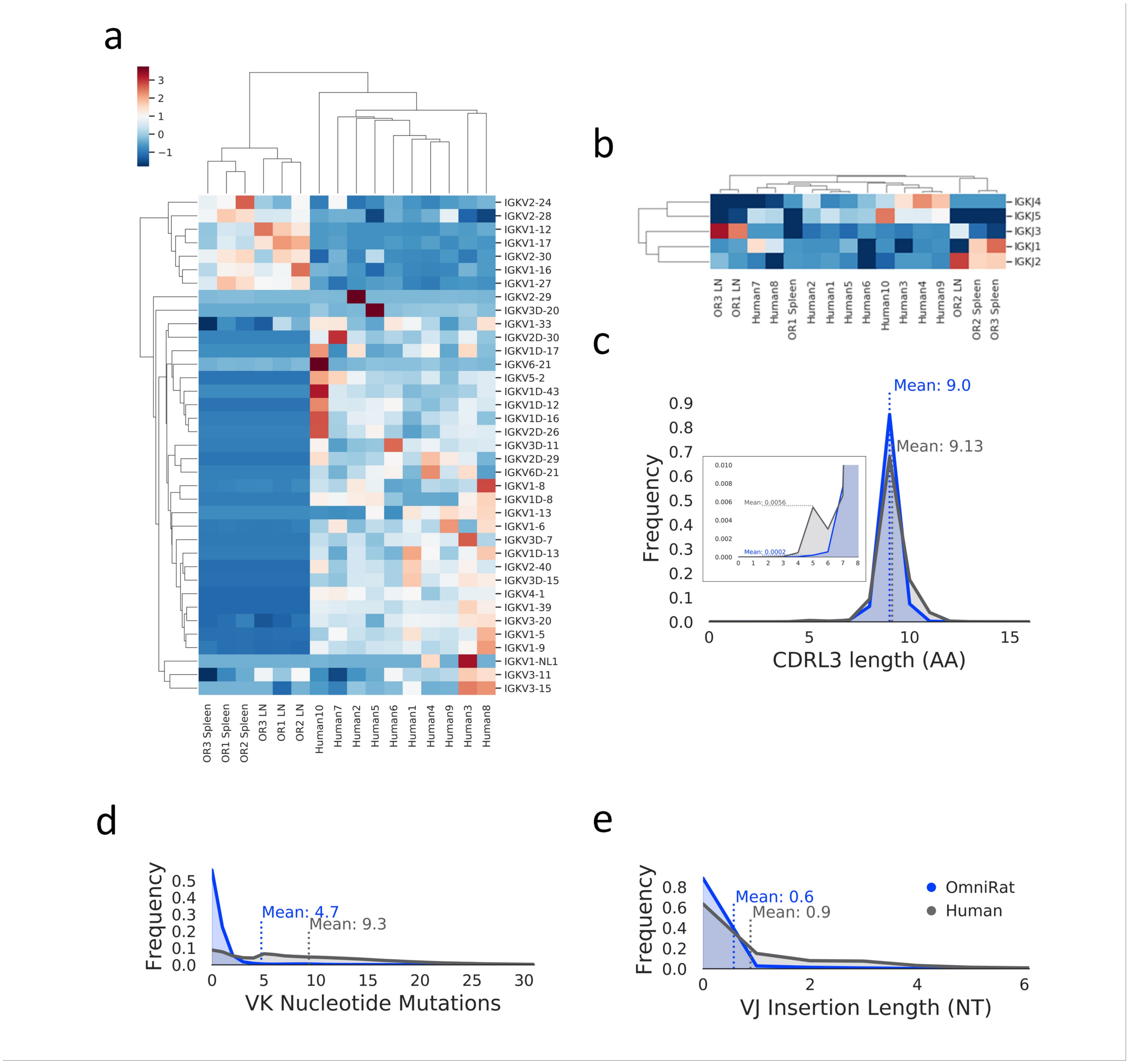

Supplementary Figure 6. Kappa chain summary. Heatmaps of variable kappa (VK) gene usage in (a) and joining kappa (JK) gene usage in (b). Columns are antibody repertoires and rows are gene segments. Data was scaled by calculating the Z-score for each gene (row) and hierarchical clustering (Euclidean distance metric) was done. A dendrogram representation of clustering is shown. c) CDRL3 length distribution for each species. Species are colored as in (e). d) Variable kappa gene nucleotide mutation number distributions. Species colored as in (e). e) VJ insertion length distributions for each species.

Animal Name	ID	Tissue	Raw Reads	Consensus Sequensus	Unique Clonotypes
OR1	15653	Lymph Node	1,977,466	902,427	209,526
		Spleen	1,583,450	1,189,591	364,080
OR2	15660	Lymph Node	2,206,738	1,240,589	321,648
		Spleen	2,392,536	1,683,726	417,943
OR3	15661	Lymph Node	3,072,548	1,703,057	391,583
		Spleen	3,895,355	2,555,076	633,635

Supplementary Table 1. OmniRat heavy chain sequencing summary.

Name	Sequence*	Step
IgM-OmniRat-RT	ACACTCTTTCCCTACACGACGCTCTTCCGATCTNNNNNNNNNNNNCAGTTGGGGAGGAC	RT
IgG-OmniRat-RT	ACACTCTTTCCCTACACGACGCTCTTCCGATCTNNNNNNNNNNNNGGATAGACAGATGG	RT

* 'N's indicate Unique Molecular Identifiers (UMIs)

Supplementary Table 2. OmniRat heavy chain reverse transcription primers.

Comparison group	Number of clonotypes
OR1, OR2, OR3	27,307
OR1, OR2	48,608
OR1, OR3	53,678
OR2, OR3	68,315
OR1	541,056
OR2	682,867
OR3	954,378

Supplementary Table 3. Number of shared clonotypes per Venn diagram comparison group from Figure 4a.
